# ASO Author Reflections: Isolated Mesoappendiceal Invasion Is Not an Indication for Right Hemicolectomy in Appendiceal Neuroendocrine Tumors

**DOI:** 10.1245/s10434-025-18150-7

**Published:** 2025-08-19

**Authors:** Diamantis I. Tsilimigras, Jordan M. Cloyd

**Affiliations:** Division of Surgical Oncology, Department of Surgery, The Ohio State University Wexner Medical Center and James Comprehensive Cancer Center, Columbus, OH USA

## Past

Current guidelines are conflicting as to whether mesoappendiceal invasion (MAI) warrants right hemicolectomy (RHC) among patients with appendiceal neuroendocrine tumors (aNET), especially in the absence of other concomitant high-risk features.^1–3^ Although MAI has long been thought to be associated with increased risk of lymph node metastasis (LNM) among patients with aNETs, most data are derived from relatively small patient populations. Therefore, the exact predictive and prognostic impact of MAI remains incompletely defined to date. In this study, we sought to characterize the probability of LNM among patients with resected aNETs in the setting of MAI alone or in combination with other high-risk features. In addition, we sought to assess the survival impact of RHC versus simple appendectomy among patients with aNET and MAI.^4^

## Present

Utilizing data from the National Cancer Database (NCDB), a total of 4819 patients who underwent aNET resection between 2018 and 2019 (limited analysis to years corresponding to 8th American Joint Committee on Cancer [AJCC] staging edition) were identified. Among them, 1662 had pT3 tumors of which 1309 (78.7%) were categorized as pT3a (< 4 cm and MAI/subserosal invasion [SI]), and 353 (21.3%) were categorized as pT3b (> 4 cm and/or MAI/SI). In the entire cohort, the overall incidence of pN+ disease was 7.5%; pT3a stage was less frequently associated with pN+ disease compared with pT3b disease (6.8% versus 14.7%, *p* = 0.02). Importantly, the presence of MAI/SI alone was associated with a low probability of pN+ (3.4%) in the absence of other high-risk factors. Among patients with MAI/SI, higher tumor size and presence of lymphovascular invasion significantly increased the risk of LNM. Among patients with aNET with definitive MAI/SI (pT3a stage), 3-year overall survival (OS) was comparable following RHC versus simple appendectomy (92.7% versus 95.2%, *p* = 0.43).

## Future

While MAI has been considered a high-risk factor for LNM among patients with aNETs, the data from the current study suggest that MAI alone, in the absence of other risk factors, is associated with a relatively low rate of LNM. In addition, RHC does not appear to offer a clear survival benefit compared with simple appendectomy in the setting of MAI alone. The data suggest that MAI/SI alone should not be an indication for RHC among patients with resected aNETs. Emerging evidence supports the safety of active surveillance after incidental aNET diagnosis, particularly in low-risk cases.^5^ Improved diagnostic modalities, including high-resolution cross-sectional and functional imaging may enhance detection of occult nodal disease and optimize patient selection for more invasive procedures. Future studies should focus on refining the indications for RHC and lymphadenectomy given the unique biology and therapeutic options of well-differentiated aNETs, ultimately aiming to minimize overtreatment for this disease.
